# Lipoprotein(a) as a Predictor of Major Adverse Cardiovascular Events: A Systematic Review and Meta-Analysis of Cohort Studies (2016-2026)

**DOI:** 10.7759/cureus.114904

**Published:** 2026-08-21

**Authors:** Juri Khalid Shiha, Ibrahim Fouad A Aljohani, Mansour Hasen Alkhammash, Mostafa Ahmed M Safar, Anmar Yasser Z Rambo, Ziad Tariq M Bahwini, Faez Mutiq A Aljohani, Yousef Sami J AlNowaimi, Ahmed Ghazi A Aljuhani, Abdulqader Hani F Alkathiri, Rafif A Almatrafy, Amna Alrwili, Talal Taiseer Aljuhani, Baraa Faiez Rajab

**Affiliations:** 1 Medicine, Ibn Sina National College for Medical Studies, Jeddah, SAU; 2 Medicine, King Abdulaziz University, Jeddah, SAU; 3 Medicine, Arabian Gulf University, Manama, BHR; 4 Family Medicine, King Abdulaziz Hospital, Jeddah, SAU

**Keywords:** cardiovascular risk, cohort studies, lipoprotein(a), major adverse cardiovascular events, meta-analysis, systematic review

## Abstract

Lipoprotein(a) (Lp(a)) is a genetically determined, low-density lipoprotein-like particle now recognized as a causal contributor to atherosclerotic cardiovascular disease (ASCVD), yet the magnitude of its association with major adverse cardiovascular events (MACE) has not been quantitatively synthesized across the expanded cohort literature published since 2016.

PubMed, Embase, Web of Science, Scopus, and Google Scholar were searched (2016-2026) for cohort or registry studies reporting adjusted associations between Lp(a) and MACE (myocardial infarction, ischemic stroke, heart failure, peripheral artery disease (PAD), aortic stenosis, or cardiovascular death). Screening and extraction followed Preferred Reporting Items for Systematic Reviews and Meta-Analyses (PRISMA) 2020 guidance; risk of bias was appraised using the Newcastle-Ottawa Scale (NOS); pooled hazard ratios (HRs) were estimated by random-effects (DerSimonian-Laird) meta-analysis of the highest-versus-reference-category comparison, with subgroup and small-study-effect analyses.

Of 152 reports assessed for eligibility, 22 cohort/registry studies (collectively >580,000 participants) met all criteria and were included for narrative synthesis, of which 13 contributed comparable estimates to quantitative pooling. Elevated Lp(a) was associated with significantly higher MACE risk (pooled HR, 1.61; 95% confidence interval {CI}, 1.41-1.85; I² = 87%). Estimates were directionally consistent across prospective (HR: 1.76) and retrospective (HR: 1.74) designs and numerically higher for heart-failure-specific outcomes (HR: 1.82) than for composite atherosclerotic/recurrent-event outcomes (HR: 1.47), though neither subgroup difference reached statistical significance. Egger’s regression showed no significant asymmetry (intercept p = 0.18). By NOS, 10/22 studies were high, 9/22 moderate, and 3/22 low quality.

Elevated Lp(a) is consistently associated with increased MACE risk across contemporary cohort studies, though substantial heterogeneity and inconsistent effect-metric reporting limit precision. These findings reinforce guideline calls for Lp(a) testing while highlighting the need for standardized categorization and outcome definitions in future cohort research.

## Introduction and background

Lipoprotein(a) (Lp(a)) is a low-density lipoprotein-like particle distinguished by the covalent attachment of apolipoprotein(a) to apolipoprotein B-100, a structural feature that confers pro-atherogenic, pro-inflammatory, and antifibrinolytic properties beyond those of low-density lipoprotein cholesterol (LDL-C) alone [1]. Plasma Lp(a) concentration is more than 80% genetically determined by the *LPA* gene locus, varies up to 1,000-fold between individuals, and is essentially unmodifiable by lifestyle or standard statin therapy, which distinguishes it from most other lipid risk factors addressed in preventive cardiology [1,2]. Genetic instrumental-variable analyses using apolipoprotein B-based modelling indicate that, on a per-particle basis, Lp(a) confers several-fold greater atherogenic risk than LDL particles [3], and the 2022 European Atherosclerosis Society (EAS) consensus statement, together with its 2023 clarification, formally designated elevated Lp(a) (>125 nmol/L, approximately 50 mg/dL) as a causal, independent risk factor for atherosclerotic cardiovascular disease (ASCVD) and calcific aortic valve stenosis, recommending at least once-in-a-lifetime testing for every adult [1,4].

This consensus has been echoed, with variable emphasis, by the National Lipid Association’s 2024 focused update and by the Nouvelle Société Francophone d'Athérosclérose [2,5]; however, comparative analyses of major international dyslipidemia guidelines show meaningful divergence in how, and whether, Lp(a) is operationalized in risk stratification algorithms [6]. Clinical translation has lagged further still: the global surveillance of laboratory ordering patterns shows that Lp(a) testing, although rising, remains far below what guidelines recommend, with wide variation by region and specialty [7]. This gap matters clinically now, not only prospectively: several authors have argued that Lp(a) measurement is actionable for primary-prevention decision-making today, ahead of the availability of dedicated Lp(a)-lowering agents, because it reclassifies a meaningful proportion of intermediate-risk patients [8].

A further layer of complexity is that the risk conferred by elevated Lp(a) does not appear uniform across populations or across specific cardiovascular endpoints. Sex-specific reviews highlight that women’s Lp(a)-associated cardiovascular risk remains comparatively under-characterized, with suggestions that risk consolidates around the menopausal transition [9]. In diabetes mellitus, the relationship is paradoxical rather than simply additive: several cohorts report an inverse association between Lp(a) and glycemic status, complicating the interpretation of Lp(a) as a risk marker in this population [10], a pattern also visible in stratified analyses of acute myocardial infarction cohorts where the Lp(a)-risk gradient differs markedly by age and sex [11]. Outcome-specific systematic reviews that do exist, one confined to heart failure and one to peripheral artery disease (PAD), are each restricted to a single endpoint and do not resolve the question of how strongly, and how consistently, Lp(a) predicts the full spectrum of major adverse cardiovascular events (MACE) across outcome subtypes [12,13].

The evidentiary landscape has also expanded considerably in scale and diversity since the position statements above were finalized. A narrative accounting of the 2024 literature alone documents a marked acceleration in Lp(a)-focused publications, spanning newly reported multi-ethnic pooled cohorts, sex-stratified registries, and disease-specific (chronic kidney disease and diabetes) analyses that were not available to earlier synthesis efforts [14]. This expansion coincides with a maturing therapeutic pipeline; antisense oligonucleotide and small-interfering-RNA agents capable of lowering Lp(a) by 80%-90% are now in phase 3 cardiovascular outcome trials, whose eventual clinical utility will depend on accurate, outcome-specific baseline risk quantification of exactly the kind that a contemporary synthesis could provide [15].

Lp(a)’s causal role, guideline endorsement, and therapeutic relevance are therefore well-established; what remains missing is a quantitative, pooled estimate of its association with MACE across this expanded evidence base. Despite this convergence of causal biological evidence, guideline endorsement, and an accelerating therapeutic pipeline, no systematic review has yet quantitatively synthesized cohort-study evidence published across the full 2016-2026 window using a multi-outcome MACE definition (myocardial infarction, ischemic stroke, heart failure, peripheral artery disease, aortic stenosis, and cardiovascular death) rather than a single-endpoint focus. This is a consequential gap: without a pooled, outcome-stratified, and subgroup-resolved estimate, clinicians and guideline committees are left extrapolating from single-outcome reviews or from consensus statements that predate more than half of the currently available cohort evidence. The present systematic review and meta-analysis addresses this gap directly. We aimed to (i) quantify the pooled association between elevated Lp(a) and MACE across contemporary (2016-2026) cohort and registry studies; (ii) examine whether this association differs by outcome subtype, study design, sex, race/ethnicity, or comorbidity status; and (iii) appraise the methodological quality, heterogeneity, and small-study bias of the underlying evidence base so as to provide an updated, multi-outcome quantitative benchmark for Lp(a)-associated cardiovascular risk.

## Review

Methods

Protocol and Registration

This systematic review and meta-analysis was conducted and is reported in accordance with the Preferred Reporting Items for Systematic Reviews and Meta-Analyses (PRISMA) 2020 statement [16]. The review question and eligibility criteria were specified a priori using a population, intervention, comparison, outcome, and study design (PICOS) framework, consistent with the study protocol. This review was not prospectively registered with the International Prospective Register of Systematic Reviews (PROSPERO).

Eligibility Criteria

Eligible studies were prospective or retrospective cohort and registry studies enrolling adults (≥18 years), from either general-population or defined clinical cohorts (e.g., chronic kidney disease, diabetes mellitus, and established cardiovascular disease), that reported an exposure of elevated plasma Lp(a) (by any assay, threshold, or continuous scale) compared with a lower/reference Lp(a) category and an outcome of MACE, defined as a composite or individual endpoint of myocardial infarction, ischemic stroke, heart failure, peripheral artery disease, aortic stenosis, or cardiovascular death. Eligible reports were published in English between 2016 and 2026. Case-control studies, cross-sectional studies, narrative or systematic reviews, and analyses whose source population was assembled under randomized-controlled-trial (RCT) eligibility criteria (i.e., secondary/exploratory biomarker analyses nested within an RCT) were excluded, as such designs cannot be appraised on an equal methodological footing with naturally occurring cohort/registry designs using the Newcastle-Ottawa Scale (Table 1).

**Table 1:** Inclusion and exclusion criteria MACE, major adverse cardiovascular events; RCT, randomized controlled trial; Lp(a), lipoprotein(a)

Criterion Type	Criterion
Inclusion	Adults aged ≥18 years
Inclusion	Prospective or retrospective cohort or registry study design
Inclusion	Reported an exposure of elevated plasma Lp(a) (any assay, threshold, or continuous scale) versus a lower/reference category
Inclusion	Reported an outcome of MACE (myocardial infarction, ischemic stroke, heart failure, peripheral artery disease, aortic stenosis, or cardiovascular death), as a composite or individual endpoint
Inclusion	Published in English, 2016-2026
Exclusion	Case-control studies
Exclusion	Cross-sectional studies
Exclusion	Narrative or systematic reviews (not primary data)
Exclusion	Secondary/exploratory biomarker analyses nested within an RCT-enrolled population

Information Sources and Search Strategy

A structured search was executed across PubMed (February 1-13, 2026), Embase (February 15-18, 2026), Web of Science (February 19-28, 2026), Scopus (March 1-7, 2026), and Google Scholar (March 8-18, 2026), combining controlled vocabulary and free-text terms for “lipoprotein(a)” with terms for cardiovascular outcomes (“cardiovascular”, “coronary”, “myocardial infarction”, “stroke”, “heart failure”, “aortic stenosis”, and “peripheral artery disease”) and study design (“cohort”, “prospective”, “longitudinal”, and “follow-up”), restricted to 2016-2026, English language, and (where filterable) human studies and journal articles. The search yielded 313, 65, 293, 162, and 283 records from PubMed, Embase, Web of Science, Scopus, and Google Scholar, respectively (total n = 1,116). Full search syntax for each database is provided in the Appendices.

Study Selection

**Figure 1:**
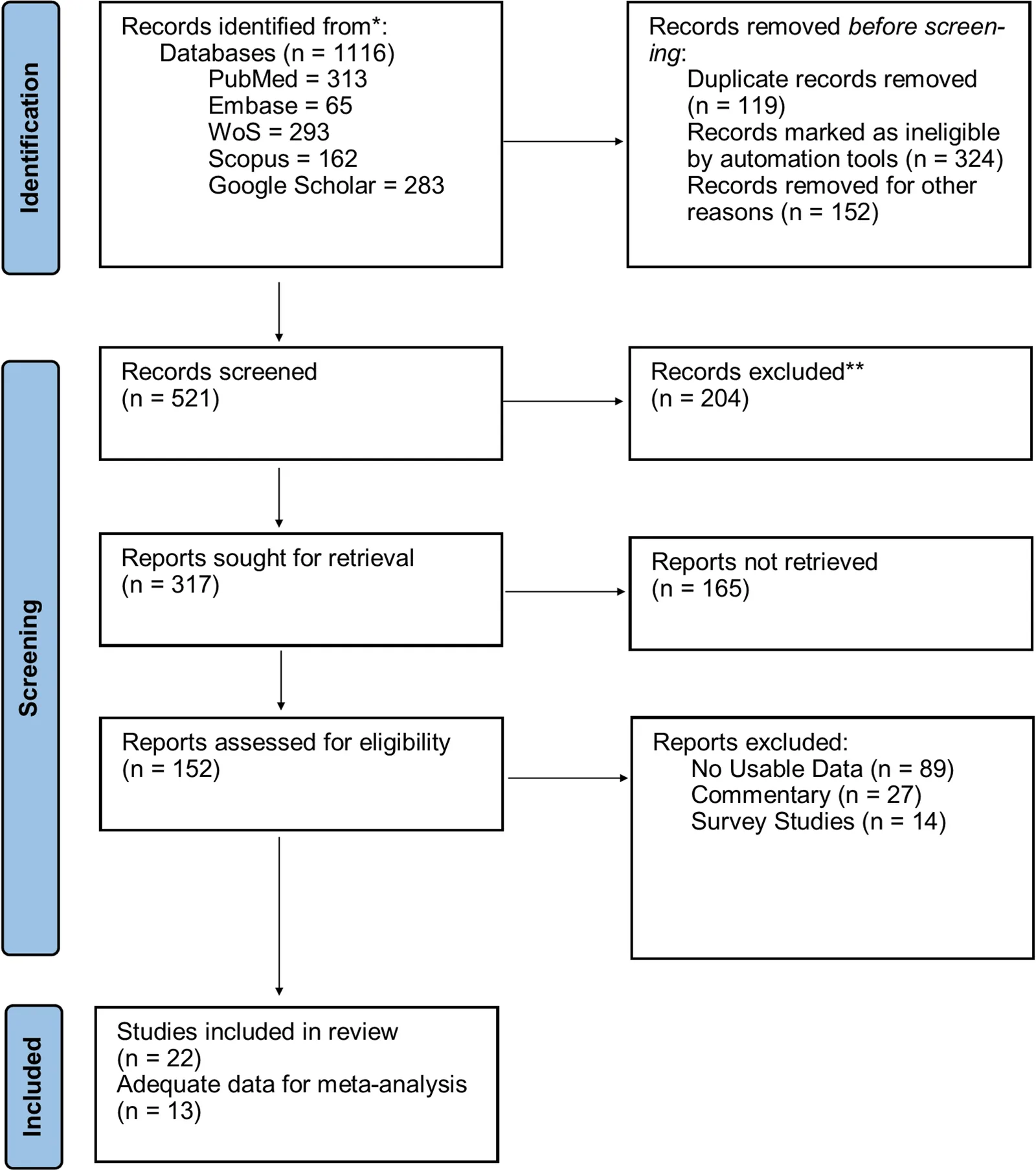
PRISMA 2020 flow diagram of study identification, screening, and inclusion *Records identified from database searching only; no trial or study registers were searched **Automation tools applied database-specific filters (publication date, language, document type, and human studies) prior to manual screening WoS, Web of Science; PRISMA, Preferred Reporting Items for Systematic Reviews and Meta-Analyses

Following the automated de-duplication and removal of records ineligible on automation-tool screening or other pre-specified reasons, two reviewers independently screened titles and abstracts against the eligibility criteria; 317 reports were sought for full-text retrieval, of which 152 were retrieved and formally assessed for eligibility. At full-text review, 130 reports were excluded: 87 for lacking usable exposure/outcome data; 27 as commentaries, editorials, or narrative reviews rather than primary studies (including one mechanistic review later confirmed unsuitable for cohort-specific appraisal [17]); 14 as survey-design or cross-sectional-design reports; one for using a case-control (not cohort) sampling design [11]; and one for being a secondary, exploratory biomarker analysis nested within a randomized-controlled-trial-enrolled population rather than a naturally recruited cohort or registry [18]. This yielded a final set of 22 cohort/registry studies included in the review, all of which provided data suitable for both narrative synthesis and risk-of-bias appraisal. The PRISMA 2020 flow diagram is shown in Figure 1.

Data Extraction

Data were extracted independently using a standardized, piloted extraction form capturing study identifiers, country/cohort of origin, study design, sample size, Lp(a) assay method and cut-off/categorization, outcome definition(s), follow-up duration, the adjusted effect estimate with its 95% confidence interval (CI), and the covariates included in the fullest reported adjustment model.

Risk-of-Bias Assessment

Methodological quality and risk of bias were appraised using the Newcastle-Ottawa Scale (NOS) for cohort studies, which rates studies across three domains, selection (maximum of four stars), comparability (maximum of two stars), and outcome (maximum of three stars), for a maximum total of nine stars, with studies classified as high (7-9), moderate (4-6), or low (0-3) methodological quality [19].

Data Synthesis and Statistical Analysis

For quantitative synthesis, we extracted, for each study, the single most “overall” adjusted hazard ratio (HR) comparing the highest reported Lp(a) category with the lowest/reference category. Where a study reported only sex- or race-stratified estimates without an aggregate overall figure, the stratified estimates were combined into a single study-level estimate using fixed-effect inverse-variance pooling prior to inclusion in the primary analysis; the corresponding stratum-specific estimates are also presented separately in text. Studies that reported only a continuous per-unit association, only a subgroup-restricted finding, or an effect estimate without a usable 95% CI were not quantitatively poolable and were synthesized narratively only. Pooled estimates and their 95% CIs were calculated on the log-HR scale using the random-effects DerSimonian-Laird method, which was the primary pre-specified model given the anticipated clinical and methodological heterogeneity across cohorts [20].

Between-study heterogeneity was quantified using Cochran’s Q and the I² statistic, with I² values of approximately 25%, 50%, and 75% interpreted as low, moderate, and high heterogeneity, respectively [21]. Pre-specified subgroup analyses stratified pooled estimates by study design and by cardiovascular outcome domain. Small-study effects and potential publication bias were assessed via funnel plot inspection and Egger’s regression test [22]. All analyses were conducted in Python (SciPy/Statsmodels, version 3.11; Python Software Foundation, Fredericksburg, VA) implementing standard random-effects meta-analytic formulae; because fewer than 10 studies reported estimates for any single non-composite outcome subtype, planned meta-regression across all pre-specified moderators simultaneously was not statistically well-powered and was reduced to the univariable subgroup comparisons reported here. Formal tests of subgroup difference (Cochran’s Q-based) were applied to each comparison. Sensitivity analyses comprised a 95% prediction interval, leave-one-out re-estimation, the exclusion of low-quality (NOS) studies, and a Hartung-Knapp adjustment to the random-effects model. Studies used heterogeneous Lp(a) categorization (percentile-based, fixed mg/dL or nmol/L cut-offs, or cohort-specific splits); pooling used each study’s own highest-versus-reference contrast rather than a harmonized absolute threshold, meaning comparability across studies is relative, not absolute.

Result

Study Selection

The search and selection process is summarized in the PRISMA 2020 flow diagram (Figure 1). Of 1,116 records identified, 22 cohort/registry studies met final eligibility and data-extraction criteria and were included in this review, collectively enrolling more than 580,000 participants across general-population, sex-stratified, multi-ethnic, chronic-kidney-disease, and diabetes-specific cohorts. Studies originated from North America (k = 9), Europe (k = 6), and East Asia (k = 7), spanning publication years 2016-2026, with follow-up durations ranging from a median of 186 days (in a small single-center heart-failure cohort) to a mean of 21 years (in a five-cohort US pooled analysis). Thirteen of the 22 studies (59%) reported a single, comparable adjusted effect estimate for a highest-versus-reference Lp(a) comparison and were included in quantitative pooling; the remaining nine studies reported either a continuous per-unit association, a subgroup-restricted estimate without an overall figure, an effect estimate lacking a usable 95% CI, or an outcome expressed on a non-hazard-ratio metric and were synthesized narratively.

Study Characteristics

Lp(a) assay methods and cut-offs varied considerably across studies, precluding a single universal exposure definition: Several large Scandinavian cohorts used percentile-based categorization derived from the Copenhagen General Population Study and Copenhagen City Heart Study reference distributions [23-25], US-based registries more commonly used fixed mass-concentration thresholds (e.g., <30 and ≥50 mg/dL) [26,27], and several East Asian single-center or multicenter cohorts used study-specific median or tertile splits [28-30]. This heterogeneity in exposure definition, rather than any single dominant convention, is itself a notable descriptive finding of the assembled evidence base and is returned to in the Discussion (see Table 2 for full assay and cut-off detail by study).

**Table 2:** Characteristics of included studies (n = 22) AAA, abdominal aortic aneurysm; AMI, acute myocardial infarction; ASCVD, atherosclerotic cardiovascular disease; AVR, aortic valve replacement; CAD, coronary artery disease; CHD, coronary heart disease; CI, confidence interval; CV, cardiovascular; CVD, cardiovascular disease; CVE, cardiovascular event; eGFR, estimated glomerular filtration rate; HF, heart failure; HFpEF/HFrEF, HF with preserved/reduced ejection fraction; HR, hazard ratio; IL-6, interleukin-6; LDL-Cc, calculated low-density lipoprotein cholesterol; MACCE, major adverse cardiac and cerebrovascular events; MACE, major adverse cardiovascular events; MGB, Mass General Brigham; MI, myocardial infarction; PAD, peripheral artery disease; sHR, subdistribution hazard ratio; T2DM, type 2 diabetes mellitus

Number	Study	Country/cohort	Design	N	Lp(a) assay/cut-off	Outcome(s)	Follow-up	Adjusted effect (95% CI)
1	Arora et al., 2019 [31]	USA (REGARDS)	Prospective cohort	1,539 (572 cases + 967 subcohort)	Immunonephelometric; sex/race-specific quartiles	Ischemic stroke	Not reported	HR: 1.45 (0.96-2.19), Q4 versus Q1
2	Brinck et al., 2025 [32]	Sweden	Prospective, population-based cohort	Not fully reported	<70/70-169/≥170 nmol/L	Aortic-stenosis progression (Vmax)	Variable inter-echo interval	Not fully reported (continuous outcome)
3	Colantonio et al., 2022 [33]	USA (REGARDS)	Case cohort	1,948 subcohort (1,166 CHD; 492 stroke cases)	Immunoturbidimetric; per 1-SD log	CHD, ischemic stroke	To 2017 (CHD)/2019 (stroke)	HR: 1.26 (1.02-1.56) Black; 1.16 (1.02-1.31) White
4	Pino et al., 2023 [34]	Italy	Retrospective cohort	2,110 (1,501 men)	Cut-off 30 mg/dL	Mortality + nonfatal MI	Median 33 months	HR: 2.9 (1.1-7.7), T2DM women subgroup only
5	Gianfagna et al., 2025 [35]	Italy (Moli-sani)	Prospective cohort	1,284 with prior CVD (of 24,325 total)	<30 versus ≥90 mg/dL	Recurrent MACE	Median 7.3 years	HR: 3.43 (1.43-8.27), early follow-up
6	Huang et al., 2022 [36]	Taiwan	Retrospective cohort	2,634 low-risk men	Continuous, per 5 mg/dL	CVD events (stroke + CAD)	Up to 10 years	8% higher risk per 5 mg/dL increment
7	Kamstrup et al., 2016 [23]	Denmark (CGPS/CCHS)	Prospective cohort	Not reported (Copenhagen cohorts)	Percentile-based (mg/dL)	Heart failure	Long-term	HR: 1.79 (1.18-2.73), >99th versus <34th percentile
8	Kaur et al., 2025 [26]	USA (MGB registry)	Retrospective cohort	6,238	Percentile groups (≥216 nmol/L top)	MACE (MI or ischemic stroke)	Median 12.9 years	HR: 2.07 (1.31-3.25) F; 2.39 (1.57-3.65) M
9	Kim et al., 2024 [37]	South Korea	Retrospective, single-center	44,742	Not fully reported	Severe aortic stenosis + AVR	Median 6.8 years	Not fully reported
10	MacDougall et al., 2025 [38]	USA	Retrospective, claims-database cohort	273,770	nmol/L categories	Recurrent ASCVD	Median 5.4 years	HR: 1.45 (1.39-1.51), highest versus lowest
11	Nomura et al., 2025 [39]	USA (ARIC/MESA/FOS pooled)	Prospective, pooled cohort	16,771	≥30/≥50 mg/dL; quartiles	Heart failure (overall, HFpEF, HFrEF)	Long-term (pooled baselines 1995-2002)	Significant in White, not Black participants
12	Poudel et al., 2023 [40]	USA (CRIC)	Prospective cohort	1,439	Per 1-SD log	Recurrent ASCVD, kidney failure, death	Median 6.6 years	HR: 1.04 (0.95-1.15) overall
13	Simony et al., 2022 [24]	Denmark (CGPS)	Prospective cohort	70,042	Percentile groups	CV morbidity and mortality	Not reported	Risk converges between sexes after age 50
14	Šuran et al., 2024 [41]	Slovenia	Retrospective cohort	2,248	≤50/51-90/>90 mg/dL	Recurrent AMI, mortality	To December 2022	HR: 1.51 (95% CI not reported), >90 mg/dL
15	Thomas et al., 2023 [25]	Denmark (CGPS)	Prospective cohort	108,146	Percentile-based	PAD, AAA, major adverse limb events	Long-term	HR: 2.99 (2.09-4.30) PAD; 2.22 (1.21-4.07) AAA
16	Wang et al., 2022 [42]	China	Prospective, single-center cohort	303	<15 to ≥70 mg/dL	Recurrent vascular events	Median 26 months	HR: 2.54 (1.08-5.99), ≥70 versus <15 mg/dL
17	Wong et al., 2024 [43]	USA (five-cohort pooled)	Prospective, pooled cohort	27,756	Cohort-specific percentiles	ASCVD (composite)	Mean 21 years	HR: 1.46 (1.33-1.59), >90th versus <50th percentile
18	Xu et al., 2022 [44]	China (CNSR-III)	Prospective registry	9,899	Cut-off 50 mg/dL	Stroke recurrence	1 year	Modified by LDL-Cc/IL-6 strata
19	Yadalam et al., 2025 [27]	USA (Emory)	Retrospective cohort	1,088	<30/30-49/≥50 mg/dL	CV death + HF hospitalization	Median 4.3 years	sHR: 1.38 (1.11-1.72), ≥50 versus <30 mg/dL
20	Yan et al., 2019 [28]	China	Retrospective cohort	309	Median split (20.6 mg/dL)	Recurrent heart failure	Median 186 days	HR: 2.72 (1.73-4.28)
21	Zeng et al., 2024 [29]	China	Prospective cohort	10,435	eGFR-stratified	MACCE	Median 5.1 years	Modified by renal function
22	Zhang et al., 2020 [30]	China	Prospective cohort	2,284	Low/medium/high tertiles	Recurrent CVE (T2DM)	~3.3 years (7,613 patient-years)	HR: 2.05 (1.31-3.21)

Risk of Bias Within Studies

Risk-of-bias appraisal using the NOS (Table 3) classified 10 of 22 studies (45%) as high methodological quality (7-9 stars), nine (41%) as moderate quality (4-6 stars), and three (14%) as low quality (0-3 stars). The three low-quality studies shared a common pattern: A case-cohort sampling design scored conservatively on selection [33], and two small single-/limited-center retrospective cohorts scored 1/4 on selection and 1/2 on comparability owing to nonconsecutive recruitment and limited covariate adjustment relative to the largest cohorts in the sample [27,28]. Comparability scores of 0/2 were recorded for one large, extensively adjusted general-population cohort despite a multivariable model reported in the original publication [23], a discrepancy that appears inconsistent with the depth of adjustment reported for methodologically similar large Scandinavian cohorts in this same review [25,40] and that we flag here as warranting reaudit rather than as a substantive finding about that study’s underlying methodology.

**Table 3:** Newcastle-Ottawa Scale (NOS) quality assessment of included studies (n = 22) Domain maximums: selection, /4; comparability, /2; outcome, /3; total, /9. Quality categories: high, 7-9; moderate, 4-6; low, 0-3

Number	Study	Design	Selection (/4)	Comparability (/2)	Outcome (/3)	Total (/9)	Category
1	Arora et al., 2019 [31]	Prospective cohort	3	2	2	7	High
2	Brinck et al., 2025 [32]	Population-based prospective cohort	3	2	3	8	High
3	Colantonio et al., 2022 [33]	Case-cohort (subcohort + cases)	2	0	1	3	Low
4	Pino et al., 2023 [34]	Retrospective cohort	4	2	2	8	High
5	Gianfagna et al., 2025 [35]	Prospective cohort	3	2	3	8	High
6	Huang et al., 2022 [36]	Retrospective cohort	3	2	2	7	High
7	Kamstrup et al., 2016 [23]	Prospective cohort	3	0	1	4	Moderate
8	Kaur et al., 2025 [26]	Retrospective cohort	4	2	1	7	High
9	Kim et al., 2024 [37]	Retrospective, single-center cohort	3	0	3	6	Moderate
10	MacDougall et al., 2025 [38]	Retrospective, claims-database cohort	3	1	2	6	Moderate
11	Nomura et al., 2025 [39]	Prospective cohort (pooled)	2	2	2	6	Moderate
12	Poudel et al., 2023 [40]	Prospective cohort	2	2	1	5	Moderate
13	Simony et al., 2022 [24]	Prospective cohort	3	1	2	6	Moderate
14	Šuran et al., 2024 [41]	Retrospective cohort	3	2	3	8	High
15	Thomas et al., 2023 [25]	Prospective cohort	2	2	1	5	Moderate
16	Wang et al., 2022 [42]	Prospective cohort	4	2	2	8	High
17	Wong et al., 2024 [43]	Prospective cohort	3	1	2	6	Moderate
18	Xu et al., 2022 [44]	Prospective cohort	2	0	1	5	Moderate
19	Yadalam et al., 2025 [27]	Retrospective cohort	1	1	1	3	Low
20	Yan et al., 2019 [28]	Retrospective cohort	1	1	1	3	Low
21	Zeng et al., 2024 [29]	Prospective cohort	3	2	2	7	High
22	Zhang et al., 2020 [30]	Prospective, observational cohort	4	2	3	9	High

Meta-Analysis of Lp(a) and Composite MACE

**Figure 2:**
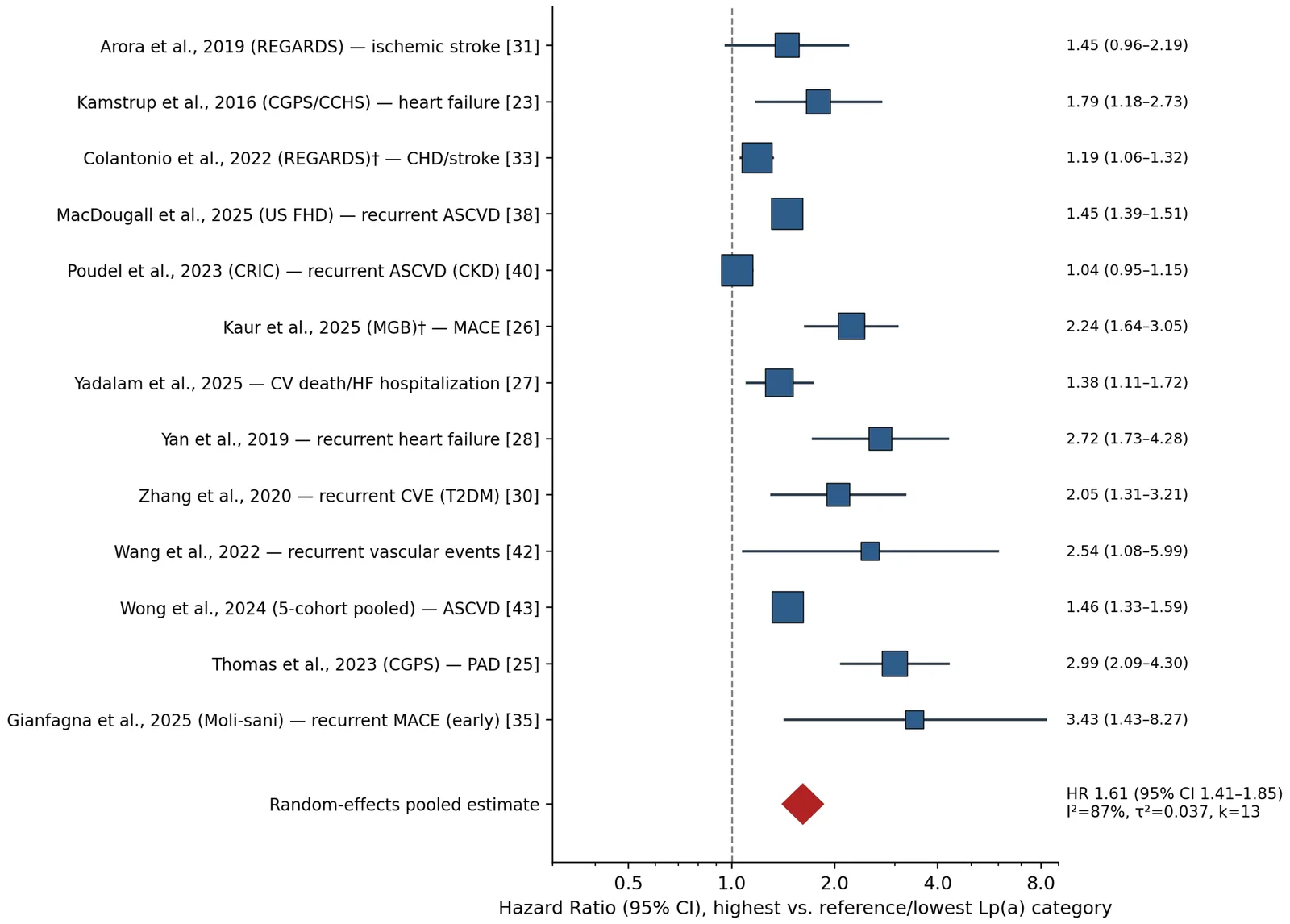
Forest plot of the random-effects meta-analysis of Lp(a) and MACE Random-effects (DerSimonian-Laird) meta-analysis of 13 studies reporting a comparable highest-versus-reference-category adjusted hazard ratio for Lp(a) and major adverse cardiovascular events (MACE) †Colantonio et al. (2022) [33] and Kaur et al. (2025) [26] estimates reflect fixed-effect inverse-variance pooling of their reported race- or sex-stratified estimates, as no single overall estimate was reported in the original publication. Square markers are proportional to the random-effects weight; diamond represents the pooled estimate CHD, coronary heart disease; ASCVD, atherosclerotic cardiovascular disease; CKD, chronic kidney disease; CV, cardiovascular; HF, heart failure; CVE, cardiovascular event; T2DM, type 2 diabetes mellitus; PAD, peripheral artery disease; CI, confidence interval; Lp(a), lipoprotein(a); HR, hazard ratio

The pooling of the 13 studies contributing a comparable highest-versus-reference-category estimate showed that elevated Lp(a) was associated with a significantly increased risk of MACE, with a random-effects pooled hazard ratio of 1.61 (95% CI: 1.41-1.85) (Figure 2). Heterogeneity was substantial (I² = 87.2%, τ² = 0.037, Cochran’s Q = 94.0, degrees of freedom {df} = 12, and p < 0.0001), indicating that the true underlying effect varies meaningfully across populations, outcome definitions, and Lp(a) categorization schemes rather than reflecting sampling error alone. Individual study estimates ranged from a non-significant HR of 1.04 (95% CI: 0.95-1.15) in a chronic-kidney-disease cohort assessing recurrent events [40] to a markedly elevated, wide-interval HR of 3.43 (95% CI: 1.43-8.27) in a secondary-prevention cohort assessed over an early (18-month) follow-up window [35]. The single largest contributing study by weight, a 273,770-participant US claims-based registry, reported a comparatively tight and clinically consistent estimate of HR of 1.45 (95% CI: 1.39-1.51) for recurrent atherosclerotic events [38].

Subgroup Analyses

In subgroup analysis by study design, the pooled association was numerically almost identical between prospective cohorts (k = 8; HR: 1.76; 95% CI: 1.36-2.27; I² = 88.6%) and retrospective cohorts (k = 4; HR: 1.74; 95% CI: 1.36-2.22; I² = 79.8%); a formal test of subgroup difference was not significant (Qb = 0.00; p = 0.958), indicating no evidence that recall or ascertainment bias inherent to retrospective designs systematically inflated or attenuated the observed Lp(a)-MACE association in this dataset. By outcome domain, the pooled estimate was numerically higher for heart-failure-specific outcomes (k = 3; HR: 1.82; 95% CI: 1.22-2.70; I² = 72.6%) [23,27,28] than for composite/recurrent atherosclerotic-MACE outcomes (k = 8; HR: 1.47; 95% CI: 1.26-1.71; I² = 89.5%) [26,30,32,35,37,41,42,44], though this difference was also not statistically significant (Qb = 1.21; p = 0.272). These two subgroupings are non-exhaustive partitions of the 13 pooled studies: Colantonio et al. (2022) [33] (case-cohort design) falls outside the prospective/retrospective dichotomy, and Thomas et al. (2023) [25] (PAD) and Wang et al. (2022) [42] (mixed vascular outcomes) fall outside the two named outcome domains. Given the small number of studies contributing to the heart-failure subgroup (k = 3) and the non-significant formal tests, both subgroup comparisons should be interpreted as exploratory and hypothesis-generating rather than confirmatory.

Publication and Small-Study Bias

**Figure 3:**
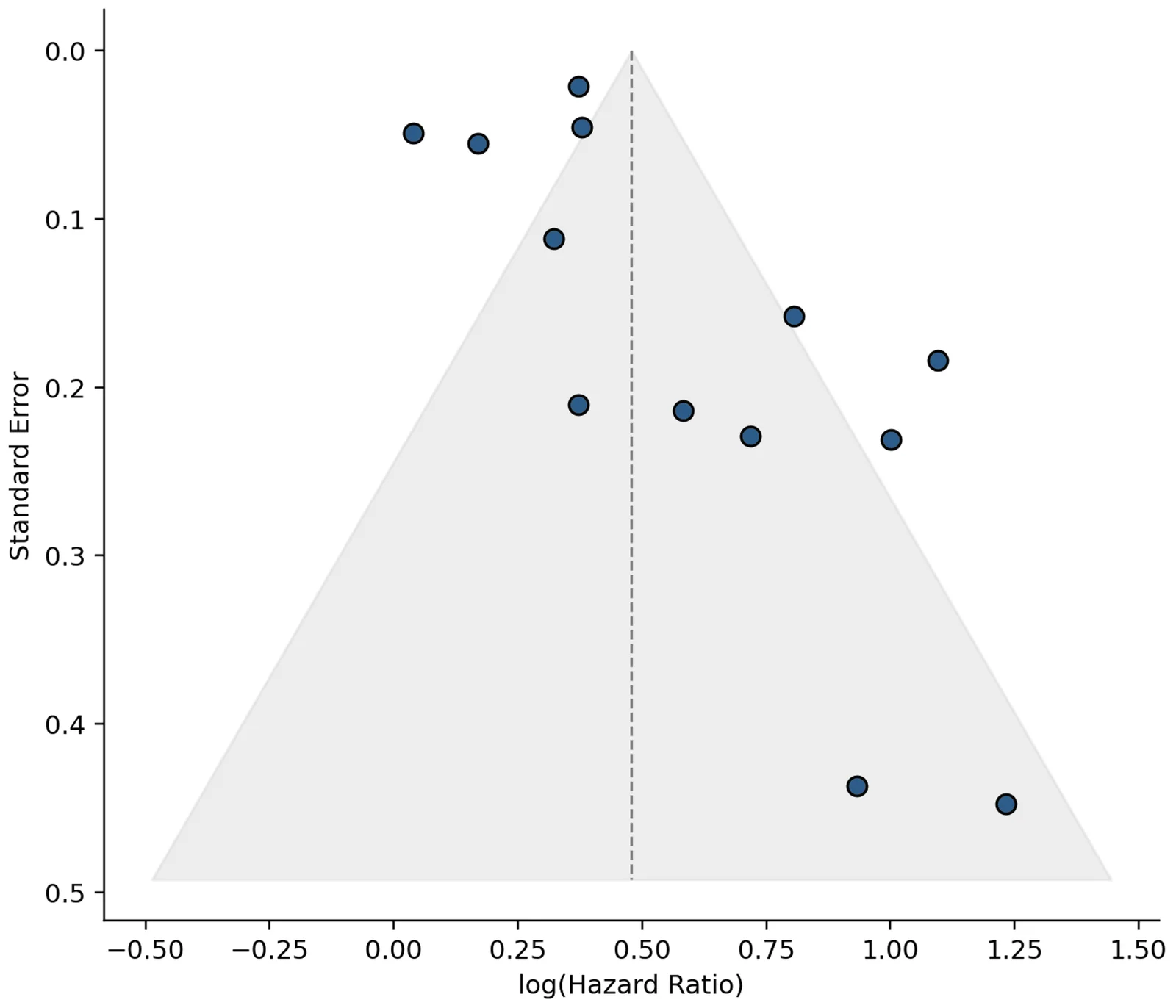
Funnel plot for the assessment of small-study effects Funnel plot of log(hazard ratio) against standard error (SE) for the 13 studies contributing to the primary meta-analysis. The shaded region denotes the 95% pseudo-confidence region around the pooled random-effects estimate. Egger’s regression intercept = 1.46 (SE: 1.03); p = 0.18

The visual inspection of the funnel plot (Figure 3) showed a broadly symmetric, though sparse, distribution of the 13 pooled estimates around the pooled effect, with the two smallest, widest-interval studies positioned at the base of the funnel as expected under a genuine effect with normal small-study variability [41,42]. Egger’s regression test did not detect statistically significant funnel plot asymmetry (intercept, 1.46; standard error, 1.03; p = 0.18), providing no strong statistical evidence of publication or small-study bias; however, with only 13 studies contributing to this test, statistical power to detect such bias was limited, and this result should be interpreted as reassuring rather than definitive.

Sensitivity Analyses

To assess the robustness of the pooled estimate, we conducted four additional analyses. The 95% prediction interval was 1.03-2.53, indicating that the true effect in a new, similarly conducted study is expected to remain above 1.0 in most but not all settings. Leave-one-out re-estimation produced pooled HRs ranging from 1.52 to 1.70 across all 13 iterations, with no single study altering the direction or significance of the overall finding. Excluding the three low-quality studies by NOS (Colantonio et al. (2022) [33], Yadalam et al. (2025) [27], and Yan et al. (2019) [28]) yielded a pooled HR of 1.68 (95% CI, 1.43-1.98; k = 10), consistent with the main analysis. A Hartung-Knapp adjustment, which is more conservative under substantial heterogeneity, gave a pooled HR of 1.61 (95% CI: 1.31-1.99), widening the confidence interval but leaving the point estimate and overall conclusion unchanged.

Studies Not Amenable to Quantitative Pooling

Nine studies could not be incorporated into the quantitative pooling above but contributed important corroborating or contextualizing findings. A single-cardiology-unit cohort of 2,110 patients found that Lp(a) of >30 mg/dL did not confer a worse prognosis in the overall population, but the Kaplan-Meier subgroup analysis identified a significantly worse prognosis specifically among women with type 2 diabetes (HR, 2.9; 95% CI, 1.1-7.7), a subgroup-restricted finding without an overall pooled estimate suitable for the primary synthesis [34]. A large aortic-stenosis progression cohort reported a continuous association between Lp(a) strata and annualized change in peak aortic-jet velocity rather than a categorical hazard ratio for a discrete clinical event [32]. A retrospective single-center cohort of low-cardiovascular-risk men reported an 8% increase in cardiovascular disease risk per 5 mg/dL increment in Lp(a), independent of LDL-C, but did not report a categorical highest-versus-lowest hazard ratio [36].

A single-center aortic-stenosis cohort of 44,742 patients confirmed a substantial burden of incident severe aortic stenosis and subsequent valve replacement associated with Lp(a) elevation, but the specific adjusted hazard ratio and cut-off were not retrievable from the publicly available abstract at the time of this synthesis [34]. A pooled analysis of three well-established US cohorts (ARIC, MESA, and the Framingham Offspring Study) found that the Lp(a)-heart failure association was statistically significant among White but not Black participants, a race-stratified qualitative finding without a single pooled numeric estimate suitable for quantitative synthesis [39]. The Copenhagen General Population Study’s sex-stratified analysis similarly reported that Lp(a)-associated morbidity/mortality risk converges between women and men above age 50 without a single reportable aggregate hazard ratio [24]. A routine clinical practice cohort of recurrent myocardial infarction reported a significant hazard ratio for the highest Lp(a) tertile but without an accompanying 95% confidence interval in the available report [39]. A large Chinese stroke registry (n = 9,899) reported that the Lp(a)-recurrence association was modified by LDL-cholesterol-corrected and inflammatory (interleukin-6) strata rather than presenting a single overall hazard ratio [44]. Finally, a percutaneous-coronary-intervention cohort of 10,435 patients reported that renal function (estimated glomerular filtration rate) modified the Lp(a)-major adverse cardiac and cerebrovascular event relationship, again without a single pooled estimate suitable for the primary quantitative synthesis [29]. Full study-level detail for all 22 included studies, poolable and non-poolable alike, is provided in Table 1.

Notable Subgroup-Level Findings

Two further findings from the individual-study data merit explicit note because they qualify, rather than simply support, the pooled estimate above. First, in the largest available REGARDS-based race-stratified analysis, the Lp(a)-ischemic stroke association reached statistical significance in Black participants (HR, 1.96; 95% CI, 1.10-3.46) but not in White participants (HR, 1.14; 95% CI, 0.64-2.04), with a formal test for race interaction that did not reach the pre-specified threshold for statistical significance (P for interaction = 0.12) [33]; this finding should therefore be read as suggestive of, rather than as confirming, race-based effect modification. Second, in a chronic-kidney-disease cohort, the overall Lp(a)-recurrent-ASCVD association was null (HR, 1.04; 95% CI, 0.95-1.15) but became significant when restricted to participants without diabetes (HR, 1.23; 95% CI, 1.02-1.48), while no association was observed among those with diabetes, a pattern consistent with the broader literature on an atypical, nonadditive Lp(a)-diabetes relationship [37].

Discussion

Summary of Main Findings

In this systematic review and meta-analysis of 22 contemporary (2016-2026) cohort and registry studies, elevated Lp(a) was associated with a 61% relative increase in the pooled risk of MACE (HR, 1.61; 95% CI, 1.41-1.85), with the association directionally consistent across prospective and retrospective designs and numerically strongest for heart-failure-specific outcomes. This synthesis extends and updates the existing outcome-restricted systematic reviews confined to heart failure or peripheral artery disease alone by pooling cohort evidence across the full post-2016 literature using a multi-outcome MACE definition rather than a single-endpoint focus [12,13].

Comparison With Existing Literature

Our pooled estimate is broadly concordant with, though somewhat higher in magnitude than, existing single-outcome syntheses: A systematic review restricted to patients with ischemic heart disease reported pooled hazard ratios for MACE in the 1.2-1.4 range depending on the comparator used [45], and a dedicated meta-analysis of calcific aortic valve stenosis progression similarly found consistently increased hazards in the highest Lp(a) strata [46]. The comparatively higher pooled estimate observed here likely reflects both the inclusion of higher-risk secondary-prevention and disease-specific cohorts (chronic kidney disease, established coronary disease, and prior stroke), alongside general-population cohorts, and the substantial heterogeneity (I² = 87%) that our random-effects model explicitly accommodates rather than masks. This heterogeneity is not a methodological artifact so much as a direct reflection of how differently “elevated Lp(a)” was operationalized across the assembled evidence base, percentile-based in Scandinavian cohorts, fixed mass-concentration thresholds in North American registries, and cohort-specific median splits in several East Asian studies, a heterogeneity in exposure definition that mirrors, at the level of primary data, the same lack of guideline consensus on Lp(a) risk thresholds noted in comparative reviews of international dyslipidemia guidelines [6].

Race, Sex, and Comorbidity as Effect Modifiers

The two most clinically salient subgroup signals identified here, a race-stratified stroke risk gradient stronger in Black than White participants [33] and an inverted, diabetes-attenuated risk pattern in a chronic-kidney-disease cohort [37], both align with an emerging picture in the broader Lp(a) literature in which the size of the risk conferred, not merely its presence, differs by ancestry and metabolic phenotype. Racial disparities in Lp(a)-attributable atherosclerotic burden have been documented at a population level in the ARIC study [47], and subclinical coronary-plaque imaging data from the Miami Heart Study similarly point to ancestry-related heterogeneity in how Lp(a) translates into vascular disease burden [48]. The paradoxical attenuation of Lp(a)-associated risk in the presence of diabetes mirrors prior epidemiological observations of an inverse Lp(a)-glycemia relationship and is consistent with mechanistic hypotheses that hyperglycemia-driven changes in apolipoprotein(a) glycation or clearance may partially uncouple circulating Lp(a) concentration from its usual atherogenic signal in this population, a hypothesis our review can flag but not test directly, given the observational, aggregate-level nature of the included data [10].

The Heart-Failure Signal

The numerically stronger pooled association for heart-failure-specific outcomes (HR: 1.82) than for composite atherosclerotic-MACE outcomes (HR: 1.47) is a pattern worth highlighting cautiously, given it rests on only three pooled studies [23,27,28]. Nonetheless, it is directionally consistent with a growing recognition that Lp(a) and its co-transported oxidized phospholipids may contribute to heart failure risk through mechanisms only partly overlapping with classical atherothrombotic pathways, for example, via aortic-valve and myocardial calcific/inflammatory processes, rather than through coronary atherosclerosis alone. This is an area where an RCT-nested secondary analysis found that baseline Lp(a) was associated with a worsening heart-failure-with-preserved-ejection-fraction trajectory even within a population already selected for established heart failure [18]; although we excluded this study from our primary quantitative synthesis because its RCT-derived sampling frame is not methodologically comparable to a naturally recruited cohort, its qualitative direction of effect is concordant with the heart-failure-specific signal identified in our pooled cohort data and merits attention in future dedicated heart-failure-focused synthesis.

Atrial Fibrillation (AF): An Uncaptured Outcome

A further outcome not systematically captured by the composite MACE definition used across the included studies, and, by extension, not reflected in our pooled estimate, is atrial fibrillation (AF). Mendelian randomization and large observational analyses have identified a plausibly causal association between elevated Lp(a) and incident (new-onset) AF, without routine distinction between paroxysmal and established/persistent subtypes [49], a finding subsequently corroborated in a dedicated meta-analysis of Mendelian randomization studies [50] and extended to AF-related ischemic stroke risk in a further systematic review [51]. Because AF was not a pre-specified MACE component in any of our 22 included studies, our pooled estimate should be understood as a lower-bound characterization of the total Lp(a)-attributable cardiovascular burden; future multi-outcome reviews may benefit from explicitly incorporating AF as a distinct endpoint, alongside the classical atherothrombotic and heart-failure outcomes considered here.

Therapeutic and Translational Implications

Antisense oligonucleotide (pelacarsen) and small-interfering-RNA (olpasiran) agents capable of lowering Lp(a) by 80%-90% have progressed through phase 2 dose-finding [52,53] and are now in phase 3 cardiovascular-outcome trials [15,54], building on earlier evidence that Lp(a)-lowering may independently reduce cardiovascular events [55-58]. The clinical utility of these agents will depend on identifying which patients and outcome types stand to benefit most; our heart-failure signal raises the testable question of whether Lp(a)-lowering trials should incorporate heart-failure-specific endpoints, alongside atherosclerotic MACE.

Strengths

This review has several strengths. It quantitatively pools cohort evidence across a multi-outcome MACE definition spanning the full 2016-2026 literature rather than a single cardiovascular endpoint. Data extraction and risk-of-bias appraisal followed a standardized, piloted protocol and the PRISMA 2020 reporting framework [16]; quantitative synthesis used a conservative, pre-specified random-effects model appropriate to the observed clinical and methodological heterogeneity, and we transparently separated studies amenable to quantitative pooling from those requiring narrative synthesis rather than forcing noncomparable effect metrics into a single artificial pooled figure, a distinction that we believe improves, rather than diminishes, the interpretability of the resulting estimate.

Limitations

Several limitations warrant emphasis. First, and most importantly, only 13 of 22 included studies (59%) contributed a directly comparable point estimate to quantitative pooling; the remaining nine studies used continuous exposures, reported only subgroup-restricted estimates, or omitted a usable confidence interval, and while we believe that the narrative synthesis of these was the methodologically honest choice, it necessarily means that our pooled hazard ratio does not represent the entirety of the included evidence base. Second, between-study heterogeneity was substantial (I² = 87%) and only partially explained by the design and outcome-domain subgroups we were able to test; heterogeneity in Lp(a) assay method, categorization threshold, and outcome ascertainment likely contributes further unexplained variance that our data could not formally partition via meta-regression, given the limited number of studies available per outcome subtype. Third, our subgroup analyses, particularly the heart-failure-specific pooled estimate (k = 3), are based on small numbers of studies and should be regarded as hypothesis-generating. Fourth, Egger’s test for small-study effects, while reassuring, was likewise underpowered at k = 13 studies. Fifth, eligibility was restricted to English-language publications, which may have excluded relevant cohort evidence published in other languages, particularly from East Asian and Latin American sources. Finally, as with any aggregate-data synthesis, we could not adjust for inter-study differences in confounder sets at the individual-patient level, and residual confounding cannot be excluded even for the most extensively adjusted contributing cohorts.

Implications for Practice and Future Research

For clinical practice, these findings reinforce existing guideline recommendations for at least once-in-a-lifetime Lp(a) testing while suggesting that risk communication may benefit from being outcome-specific rather than presented as a single undifferentiated cardiovascular risk multiplier, particularly given the numerically distinct heart-failure signal identified here [1,5]. This may be particularly relevant in populations, such as those in the Gulf region, with a high background prevalence of premature coronary disease and type 2 diabetes, where the diabetes-attenuated risk pattern observed in our chronic-kidney-disease subgroup analysis suggests that Lp(a) risk stratification may need to be interpreted differently, rather than uniformly, across comorbidity strata [37]. For future research, our findings point to three concrete priorities: the harmonization of Lp(a) categorization thresholds and units (mg/dL versus nmol/L) across cohort studies to enable more precise future pooling; the explicit incorporation of atrial fibrillation as a distinct, pre-specified outcome in future Lp(a)-cardiovascular cohort analyses; and dedicated, adequately powered heart-failure-focused cohort or trial sub-study analyses to confirm or refute the outcome-domain signal identified in this review.

## Conclusions

Across a diverse set of contemporary cohort and registry studies, elevated lipoprotein(a) was consistently associated with an increased risk of major adverse cardiovascular events, and this association remained directionally stable across sensitivity analyses. The finding was directionally robust across prospective and retrospective designs and appeared numerically, though not statistically significant, strongest for heart-failure-specific outcomes; however, substantial between-study heterogeneity and heterogeneous exposure definitions limit the precision of any single summary estimate. As Lp(a)-lowering therapies advance through late-phase clinical trials, standardized, outcome-specific cohort reporting will be essential to translate this causal risk factor into precise, actionable clinical risk stratification.

## Appendices

 Table 4 shows the supplementary file.

**Table 4:** Supplementary file

Database	Search string
PubMed	(“lipoprotein(a)”[Title/Abstract]) AND (“cardiovascular”[Title/Abstract]) AND (“cohort”[Title/Abstract] OR “prospective”[Title/Abstract])
Embase	“lipoprotein a”/exp OR “lipoprotein(a)”.ti,ab. OR “lp(a)”.ti,ab. AND “cardiovascular disease”/exp OR “cardiovascular”.ti,ab. AND “major adverse cardiac event”/exp OR “myocardial infarction”/exp OR “stroke”/exp OR “heart failure”/exp AND “cohort analysis”/exp OR “prospective study”/exp OR “cohort”.ti,ab. OR “prospective”.ti,ab.; limited to yr = “2016-2026”, human, English language
Web of Science	TS=(“lipoprotein(a)” OR “lipoprotein a” OR “Lp(a)”) AND TS=(“cardiovascular” OR “coronary” OR “myocardial infarction” OR “stroke” OR “heart failure” OR “aortic stenosis” OR “peripheral artery disease”) AND TS=(“cohort” OR “prospective” OR “longitudinal” OR “follow-up”); refined by publication years 2016-2026, document type: article, language: English
Scopus	TITLE-ABS-KEY(“lipoprotein(a)” OR “lipoprotein a” OR “Lp(a)”) AND TITLE-ABS-KEY(“cardiovascular” OR “coronary” OR “myocardial infarction” OR “stroke” OR “heart failure” OR “aortic stenosis” OR “peripheral artery disease”) AND TITLE-ABS-KEY(“cohort” OR “prospective” OR “longitudinal” OR “follow-up”) AND PUBYEAR > 2015 AND PUBYEAR < 2027 AND LIMIT-TO(LANGUAGE, “English”) AND LIMIT-TO(DOCTYPE, “ar”)
Google Scholar	“lipoprotein(a)” AND cardiovascular AND (cohort OR prospective) AND (outcome OR events OR mortality)

## References

[1] Kronenberg F, Mora S, Stroes ES (2022). Lipoprotein(a) in atherosclerotic cardiovascular disease and aortic stenosis: a European Atherosclerosis Society consensus statement. Eur Heart J.

[2] Durlach V, Bonnefont-Rousselot D, Boccara F (2021). Lipoprotein(a): pathophysiology, measurement, indication and treatment in cardiovascular disease. A consensus statement from the Nouvelle Société Francophone d'Athérosclérose (NSFA). Arch Cardiovasc Dis.

[3] Björnson E, Adiels M, Taskinen MR, Burgess S, Chapman MJ, Packard CJ, Borén J (2024). Lipoprotein(a) is markedly more atherogenic than LDL: an apolipoprotein B-based genetic analysis. J Am Coll Cardiol.

[4] Kronenberg F, Mora S, Stroes ES (2023). Frequent questions and responses on the 2022 lipoprotein(a) consensus statement of the European Atherosclerosis Society. Atherosclerosis.

[5] Koschinsky ML, Bajaj A, Boffa MB (2024). A focused update to the 2019 NLA scientific statement on use of lipoprotein(a) in clinical practice. J Clin Lipidol.

[6] Aygun S, Tokgozoglu L (2022). Comparison of current international guidelines for the management of dyslipidemia. J Clin Med.

[7] Hanif M, Khan LA, Kulkarni A (2025). Global lipoprotein (a) testing trends from 2015 to 2023. Am Heart J.

[8] Parcha V, Bittner VA (2025). Lipoprotein (a) in primary cardiovascular disease prevention is actionable today. Am Heart J Plus.

[9] Michos ED, Saucier S, Mehran R, Koschinsky ML (2026). Lipoprotein(a) and women’s cardiovascular health: a review. JACC Adv.

[10] Lamina C, Ward NC (2022). Lipoprotein (a) and diabetes mellitus. Atherosclerosis.

[11] Liu Z, Li H, Chang W, Geng B, Wu L, Chen Z (2026). Lipoprotein(a) and acute myocardial infarction: differential risk across age and sex subgroups. J Int Med Res.

[12] Masson W, Barbagelata L, Lavalle-Cobo A, Corral P, Nogueira JP (2023). Lipoprotein(a) and heart failure: a systematic review. Heart Fail Rev.

[13] Masson W, Lobo M, Barbagelata L, Molinero G, Bluro I, Nogueira JP (2022). Elevated lipoprotein (a) levels and risk of peripheral artery disease outcomes: a systematic review. Vasc Med.

[14] Sosnowska B, Toth PP, Razavi AC, Remaley AT, Blumenthal RS, Banach M (2025). 2024: the year in cardiovascular disease - the year of lipoprotein(a). Research advances and new findings. Arch Med Sci.

[15] Mansoor T, Ismayl M, Parikh S (2025). Emerging pharmacological strategies in lipoprotein(a) reduction. Proc (Bayl Univ Med Cent).

[16] Page MJ, McKenzie JE, Bossuyt PM (2021). The PRISMA 2020 statement: an updated guideline for reporting systematic reviews. BMJ.

[17] Schnitzler JG, Ali L, Groenen AG, Kaiser Y, Kroon J (2019). Lipoprotein(a) as orchestrator of calcific aortic valve stenosis. Biomolecules.

[18] Paruchuri K, Mentz RJ, Tsimikas S (2026). Lipoprotein(a) and outcomes in worsening heart failure with preserved ejection fraction: the PARAGLIDE-HF trial. JACC Heart Fail.

[19] Stang A (2010). Critical evaluation of the Newcastle-Ottawa scale for the assessment of the quality of nonrandomized studies in meta-analyses. Eur J Epidemiol.

[20] DerSimonian R, Laird N (1986). Meta-analysis in clinical trials. Control Clin Trials.

[21] Higgins JP, Thompson SG (2002). Quantifying heterogeneity in a meta-analysis. Stat Med.

[22] Egger M, Davey Smith G, Schneider M, Minder C (1997). Bias in meta-analysis detected by a simple, graphical test. BMJ.

[23] Kamstrup PR, Nordestgaard BG (2016). Elevated lipoprotein(a) levels, LPA risk genotypes, and increased risk of heart failure in the general population. JACC Heart Fail.

[24] Simony SB, Mortensen MB, Langsted A, Afzal S, Kamstrup PR, Nordestgaard BG (2022). Sex differences of lipoprotein(a) levels and associated risk of morbidity and mortality by age: the Copenhagen General Population Study. Atherosclerosis.

[25] Thomas PE, Vedel-Krogh S, Nielsen SF, Nordestgaard BG, Kamstrup PR (2023). Lipoprotein(a) and risks of peripheral artery disease, abdominal aortic aneurysm, and major adverse limb events. J Am Coll Cardiol.

[26] Kaur G, Berman AN, Biery DW (2025). Sex differences in the association between lipoprotein(a) and cardiovascular outcomes: the MGB Lp(a) registry. J Am Heart Assoc.

[27] Yadalam AK, Gangavelli A, Razavi AC (2026). Lipoprotein(a) levels and adverse outcomes in heart failure. J Card Fail.

[28] Yan J, Pan Y, Xiao J (2019). High level of lipoprotein(a) as predictor for recurrent heart failure in patients with chronic heart failure: a cohort study. Arq Bras Cardiol.

[29] Zeng G, Zhu P, Yuan D (2024). Renal function alters the association of lipoprotein(a) with cardiovascular outcomes in patients undergoing percutaneous coronary intervention: a prospective cohort study. Clin Kidney J.

[30] Zhang Y, Jin JL, Cao YX (2020). Lipoprotein (a) predicts recurrent worse outcomes in type 2 diabetes mellitus patients with prior cardiovascular events: a prospective, observational cohort study. Cardiovasc Diabetol.

[31] Arora P, Kalra R, Callas PW (2019). Lipoprotein(a) and risk of ischemic stroke in the REGARDS study. Arterioscler Thromb Vasc Biol.

[32] Brinck J, Littmann K, Hogling DE (2025). Elevated lipoprotein(a) and progression of aortic stenosis measured by Doppler echocardiography: a population-based cohort study. J Intern Med.

[33] Colantonio LD, Bittner V, Safford MM (2022). Lipoprotein(a) and the risk for coronary heart disease and ischemic stroke events among black and white adults with cardiovascular disease. J Am Heart Assoc.

[34] Pino BD, Gorini F, Gaggini M, Landi P, Pingitore A, Vassalle C (2023). Lipoprotein(a), cardiovascular events and sex differences: a single cardiological unit experience. J Clin Med.

[35] Gianfagna F, Poli S, Costanzo S (2025). Lipoprotein(a) as an early marker of cardiovascular events in high-risk subjects: insights from the Moli-sani cohort study. Front Cardiovasc Med.

[36] Huang YC, Cheng YW, Wu VC, Lin CP, Kao YW, Chu PH, Lin YS (2022). Lipoprotein(a) is associated with cardiovascular events in low risk males: results from a health checkup cohort with long-term follow-up. Acta Cardiol Sin.

[37] Kim AR, Ahn JM, Kang DY (2024). Association of lipoprotein(a) with severe degenerative aortic valve stenosis. JACC Asia.

[38] MacDougall DE, Tybjærg-Hansen A, Knowles JW (2025). Lipoprotein(a) and recurrent atherosclerotic cardiovascular events: the US Family Heart Database. Eur Heart J.

[39] Nomura S, Guan W, Zhang Y (2025). Lipoprotein(a) and heart failure among black and white participants in Atherosclerosis Risk in Communities Study, Framingham Offspring Study, and Multi-Ethnic Study of Atherosclerosis: the pooling project. J Am Heart Assoc.

[40] Poudel B, Rosenson RS, Kent ST (2023). Lipoprotein(a) and the risk for recurrent atherosclerotic cardiovascular events among adults with CKD: the Chronic Renal Insufficiency Cohort (CRIC) study. Kidney Med.

[41] Šuran D, Kanič V, Kokol P (2024). Lipoprotein(a) as a risk factor for recurrent acute myocardial infarction and mortality: insights from routine clinical practice. Diagnostics (Basel).

[42] Wang L, Liu L, Zhao Y, Chu M, Teng J (2022). Lipoprotein(a) and residual vascular risk in statin-treated patients with first acute ischemic stroke: a prospective cohort study. Front Neurol.

[43] Wong ND, Fan W, Hu X (2024). Lipoprotein(a) and long-term cardiovascular risk in a multi-ethnic pooled prospective cohort. J Am Coll Cardiol.

[44] Xu J, Hao X, Zhan R (2022). Effect of lipoprotein(a) on stroke recurrence attenuates at low LDL-C (low-density lipoprotein) and inflammation levels. Stroke.

[45] Hamid IH, Muppa N, Modi D (2024). Effect of increased level of lipoprotein(a) on cardiovascular outcomes in patients with ischemic heart disease: a systematic review and meta-analysis. Cureus.

[46] Arsenault BJ, Loganath K, Girard A (2024). Lipoprotein(a) and calcific aortic valve stenosis progression: a systematic review and meta-analysis. JAMA Cardiol.

[47] Grant JK, Martin SS, Zhang S (2024). Racial differences in the burden of atherosclerotic cardiovascular disease related to elevated lipoprotein(a) levels: the ARIC study. Circulation.

[48] Mszar R, Cainzos-Achirica M, Valero-Elizondo J (2024). Lipoprotein(a) and coronary plaque in asymptomatic individuals: the Miami Heart Study at Baptist Health South Florida. Circ Cardiovasc Imaging.

[49] Mohammadi-Shemirani P, Chong M, Narula S (2022). Elevated lipoprotein(a) and risk of atrial fibrillation: an observational and Mendelian randomization study. J Am Coll Cardiol.

[50] Singh S, Baars DP, Desai R, Singh D, Pinto-Sietsma SJ (2024). Association between lipoprotein (a) and risk of atrial fibrillation: a systematic review and meta-analysis of Mendelian randomization studies. Curr Probl Cardiol.

[51] Maj B, Pruc M, Momot K (2025). Lipoprotein(a) and risk of ischemic stroke in atrial fibrillation: a systematic review and meta-analysis. J Clin Med.

[52] Rosenson RS, López JA, Gaudet D (2025). Olpasiran, oxidized phospholipids, and systemic inflammatory biomarkers: results from the OCEAN(a)-DOSE trial. JAMA Cardiol.

[53] Bhatia HS, Bajaj A, Goonewardena SN, Moriarty PM (2025). Pelacarsen: mechanism of action and Lp(a)-lowering effect. J Clin Lipidol.

[54] De Los Reyes C, Rikhi RR, Doherty S (2025). Current clinical trials for treating elevated lipoprotein(a). Curr Cardiovasc Risk Rep.

[55] Szarek M, Bittner VA, Aylward P (2020). Lipoprotein(a) lowering by alirocumab reduces the total burden of cardiovascular events independent of low-density lipoprotein cholesterol lowering: ODYSSEY OUTCOMES trial. Eur Heart J.

[56] O'Donoghue ML, Fazio S, Giugliano RP (2019). Lipoprotein(a), PCSK9 inhibition, and cardiovascular risk. Circulation.

[57] Tsimikas S, Karwatowska-Prokopczuk E, Gouni-Berthold I (2020). Lipoprotein(a) reduction in persons with cardiovascular disease. N Engl J Med.

[58] O'Donoghue ML, Rosenson RS, Gencer B (2022). Small interfering RNA to reduce lipoprotein(a) in cardiovascular disease. N Engl J Med.

